# Meta-synthesis of qualitative studies on home-based exercise rehabilitation experiences among stroke patients: a continuity of care perspective

**DOI:** 10.3389/fresc.2026.1742902

**Published:** 2026-03-04

**Authors:** Xinxin Xu, Huangling Xiao, Minghui Zhang, Pici Li, Linlin Fang

**Affiliations:** 1 Department of Nursing, Cangnan County Hospital of Traditional Chinese Medicine, Wenzhou, Zhejiang, China; 2 Department of Nursing, Zhongshan Hospital Affiliated to Fudan University, Xuhui District, Shanghai, China

**Keywords:** continuity of care, exercise rehabilitation experience, meta-synthesis, qualitative research, stroke

## Abstract

**Background:**

Home-based exercise rehabilitation plays a vital role in enhancing functional recovery among stroke patients; however, adherence remains low because of limited continuity of care, inadequate professional supervision, and discontinuities in the transition from home to community settings. Although previous studies have examined home-based rehabilitation experiences, they lack systematic integration from the perspective of care continuity, which hinders the development of a collaborative intervention framework across different care settings.

**Purpose:**

This study synthesizes the real-world experiences of stroke patients transitioning from hospital to home rehabilitation. It aims to elucidate how care continuity influences rehabilitation behaviors and to provide insights for optimizing nursing strategies.

**Method:**

A comprehensive search of six major databases (including PubMed, Embase, and CINAHL) was conducted. Ten qualitative studies were identified, appraised, and synthesized following the Joanna Briggs Institute (JBI) framework.

**Result:**

The synthesis included 263 stroke patients. Four major categories were identified: (1) deficiencies in discharge guidance and follow-up; (2) challenges in self-regulation and home-based motivation; (3) the facilitating role of family and community support; and (4) the influence of nursing and family support continuity. Existing services exhibit marked transitional discontinuities.

**Conclusion:**

The home rehabilitation experience is shaped by the interplay among care continuity, self-regulation, and support networks. We propose a closed-loop model encompassing guidance, monitoring, and motivation. This framework provides a practical approach to optimizing the hospital-community-home collaborative system, thereby promoting sustained adherence and improving long-term recovery outcomes.

**Systematic Review Registration:**

https://www.crd.york.ac.uk/PROSPERO/view/CRD42023405581, PROSPERO CRD42023405581.

## 1 Introduction

Stroke has emerged as one of the leading chronic diseases contributing to the global burden of mortality and disability. According to the Global Burden of Disease Study 2023 (GBD 2023), the global prevalence of stroke is estimated at approximately 156.5 million (1), with more than 70% of survivors experiencing residual impairments, including gait and balance disorders, joint contractures, and motor dysfunction (2–4). These sequelae result in reduced quality of life and increased care dependency, effectively transforming stroke from an acute, life-threatening event into a chronic condition with enduring effects on functional capacity and overall quality of life (5). A substantial proportion of patients continue to experience residual upper limb impairments, gait difficulties, fatigue, and emotional challenges upon discharge, entering a functional vulnerability period characterized by limited professional support (3).This phase of exercise rehabilitation exhibits both high plasticity and considerable vulnerability. Although home and community environments can offer patients frequent, low-cost daily training opportunities, the absence of timely guidance and monitoring results in a continued decline in training adherence six weeks after discharge (6), directly affecting patients' societal reintegration, risk of relapse, and care burden (3, 7). Consequently, converting potential rehabilitation opportunities into sustained out-of-hospital rehabilitation behaviours constitutes a critical nursing challenge significantly influencing patients' long-term outcomes.

The post-discharge phase represents a pivotal point in the stroke rehabilitation continuum. Extensive evidence suggests that home-based rehabilitation provides notable advantages regarding cost-effectiveness, accessibility, and individualized support (8–11). Overall, home-based rehabilitation achieves outcomes comparable to inpatient programs in enhancing motor function and reducing readmission and fall rates (12, 13), rendering it especially suitable for patients in the subacute and chronic phases. Nevertheless, adherence to home-based rehabilitation remains suboptimal, with only about 17.8% to 62% of patients consistently completing prescribed exercises (14, 15). Significant barriers encompass the lack of professional guidance and supervision, inadequate incentive mechanisms, and patients' limited comprehension of the objectives and effectiveness of training (16). Moreover, the effectiveness of home-based rehabilitation is influenced not only by individual capacity but also by multiple contextual factors, including the home environment, caregiver competence, social support, and continuity of care services (17, 18).

Notably, continuity of care, follow-up, and support led by nurses through collaboration across multiple levels are essential for bridging this transitional gap (19). In a Chinese economic evaluation, a rehabilitation program delivered by nurses in patients' homes effectively improved both quality of life and quality-adjusted life years (QALY) among stroke patients (20). Moreover, interventions led by nurses play a key role in encouraging stroke survivors to adhere to rehabilitation plans in the home setting (21). Follow-up visits, telephone supervision, and remote monitoring can substantially improve patients adherence to exercise programs and their sense of psychological security (22, 23). At the same time, the interaction between motor function rehabilitation training and family involvement can effectively enhance patients motor performance and overall quality of life (24). From a theoretical perspective, based on continuity of care theory, nurses' guidance through skill training, monitoring via remote follow-up, and motivation through goal setting form a dynamic equilibrium within patients rehabilitation experiences (18). Social support and peer interaction can strengthen behavioural persistence via social identification and emotional resonance, with nurses acting as coordinators of support networks (18, 25). This suggests that continuity of care models led by nurses represent technical interventions and long-term care mechanisms founded on relationships, communication, and feedback.

Quantitative studies have documented the widespread issue of low adherence (14, 15) but have not thoroughly explored the underlying complex mechanisms. In recent years, qualitative research has increasingly examined the influence of nurses on stroke patients experiences with home exercise rehabilitation, yet the findings remain fragmented. Accordingly, this study utilizes a meta-synthesis approach (26) within qualitative research, adopting a distinct perspective on nursing continuity. It systematically integrates findings from diverse contexts to reveal the facilitators and barriers influencing home exercise rehabilitation experiences for stroke patients during the transition from hospital to home. The study aims to elucidate the synergistic mechanisms between nursing support and family and community support systems, ultimately developing an integrated support framework in which nurses serve as central connectors.

## 2 Materials and methods

### 2.1 Research design

This qualitative synthesis study follows the ENTREQ (Enhancing Transparency in Qualitative Research Synthesis) (27) and PRISMA (Preferred Reporting Items for Systematic Reviews and Meta Analyses) (28) guidelines. The study protocol has been registered with PROSPERO (Registration Number: CRD42023405581).

### 2.2 Search strategy

The literature search was performed per the Joanna Briggs Institute (JBI) Methodology Guide, following a three-step procedure. Step 1 comprised an initial search of the PubMed and CINAHL (EBSCO) databases, examining terms in titles, abstracts, and subject headings. Step 2 comprised comprehensive searches in PubMed, Cochrane Library, Embase, CINAHL (EBSCO), Web of Science, and PsycINFO (EBSCO) using a combination of subject terms and free-text words. Step 3 involved snowball searches by reviewing the reference lists of included studies to identify additional relevant research. The search period extended from the inception of each database to October 19, 2025. The search focused on qualitative studies exploring stroke patients' experiences with home exercise rehabilitation. Detailed search strategies are presented in Supplementary File S1, Table S1.

### 2.3 Inclusion and exclusion criteria

The Population, Phenomenon of Interest, Context, and Study Design (PICoS) framework recommended by the Australian JBI Center for Evidence-Based Healthcare established inclusion and exclusion standards per the study's primary focus. Inclusion criteria: (1) Study subjects: Patients with a confirmed diagnosis of ischemic or hemorrhagic stroke, encompassing the acute, recovery, and sequelae phases. (2) Phenomenon of Interest: Stroke patients' subjective experiences during exercise rehabilitation, including key elements such as physical and psychological sensations during training, motivation for adherence, barriers and challenges, needs and expectations, and perceived outcomes. (3) Context: Exercise rehabilitation conducted in authentic home or community settings, including stroke units, rehabilitation institutions, community rehabilitation centers, outpatient clinics, and home environments. (4) Research Design: Original studies employing explicit qualitative methods, including phenomenology, grounded theory, ethnography, or thematic analysis, and mixed-methods studies from which qualitative data can be independently extracted. Exclusion criteria: Non-English literature; studies with inaccessible full texts, incomplete data, or missing critical information; mixed-method studies in which qualitative content is highly confounded with quantitative data and cannot be extracted separately; and duplicate publications.

### 2.4 Literature screening and data extraction

Two researchers (XX and HX), trained in evidence-based methods and qualitative research, conducted literature screening and data extraction independently. Any discrepancies were resolved through consultation with a third researcher (MZ). The process included the following steps: (1) Duplicate records were removed using EndNote literature management software; (2) The researchers (XX and HX) independently assessed titles and abstracts against PICos criteria to identify documents for full-text review; (3) Full-text examination determined final inclusion.

### 2.5 Quality evaluation

Two researchers (XX and HX) independently assessed the included studies using the JBI Critical Appraisal Checklist for Qualitative Research (29). The checklist comprises 10 items, each rated as Yes (Y), No (N), or Unclear (U). Studies were included in the meta-synthesis if six or more of the 10 criteria were rated Yes. Any evaluation discrepancies were resolved through discussion with a third researcher (MZ).

### 2.6 Data extraction

Two researchers (XX and HX) independently extracted data using a standardized tool based on the JBI Qualitative Research Systematic Meta Synthesis Design (30). To address the specific requirements of the study on stroke patients'experiences with exercise rehabilitation, they added dimensions such as nursing and rehabilitation professional involvement, scenario type, and post-stroke timeframe. Any discrepancies were resolved by a third researcher (MZ). Extracted content included study basic information (authors, year, country or region, study design), sample characteristics (sample size, mean age, female proportion, post-stroke time window), methodology (primary analytical methods), and core themes (research focus and main themes).

### 2.7 Data synthesis

The results of the included studies were meta-synthesized using the System for the Unified Management, Assessment and Review of Information (SUMARI), a web-based systematic review and meta-analysis tool developed by JBI (31), following these steps: (1) One researcher (XX) entered the results, while another researcher (MZ) conducted a review; (2) Two researchers (XX and HX) independently summarized findings into individual statements and assigned credibility ratings of clear, plausible, or lacking support to ensure consistency between citations and findings; (3) Findings rated clear or plausible were categorized by content, while those rated lacking support were excluded from further analysis; (4) Categories were subsequently consolidated into comprehensive findings. All authors reviewed the appropriateness of these findings, and any disagreements were resolved through discussion with a third researcher (MZ). To enhance transparency, the synthesis process followed the standard JBI meta-aggregation hierarchy: primary findings were extracted from the original qualitative data, grouped into conceptual categories based on similarity, and finally integrated into overarching synthesized findings to inform clinical practice.

### 2.8 Credibility of research findings

The ConQual evidence grading method (32) was employed to assess the quality of evidence in the integrated findings. All integrated findings were initially considered high quality and downgraded based on dependability and credibility ratings, ultimately establishing the final evidence quality level.

#### 2.8.1 Dependability rating

Dependability was rated based on the evaluation of items 2, 3, 4, 6, and 7 of the JBI Critical Appraisal Checklist for Qualitative Research. Items 2–4 relate to methodological congruity (alignment between research questions, methods, data collection, and analysis), whereas Items 6–7 address reflexivity and researcher influence. If four or five items are rated Yes, the rating remains unchanged; if two or three items are rated Yes, the rating is downgraded by one level; and if zero or one item is rated Yes, the rating is downgraded by two levels.

#### 2.8.2 Credibility assessment

If the integrated results include only explicit original research findings, the rating remains unchanged; if they include both explicit and plausible findings, the rating is downgraded by one level; if they include only plausible findings, the rating is downgraded by one level; if they include only reasonable findings, the rating is downgraded by two levels; if they include findings lacking support, the rating is downgraded by three levels; if all findings lack support, the rating is downgraded by four levels.

## 3 Result

### 3.1 Literature search results

**Figure 1:**
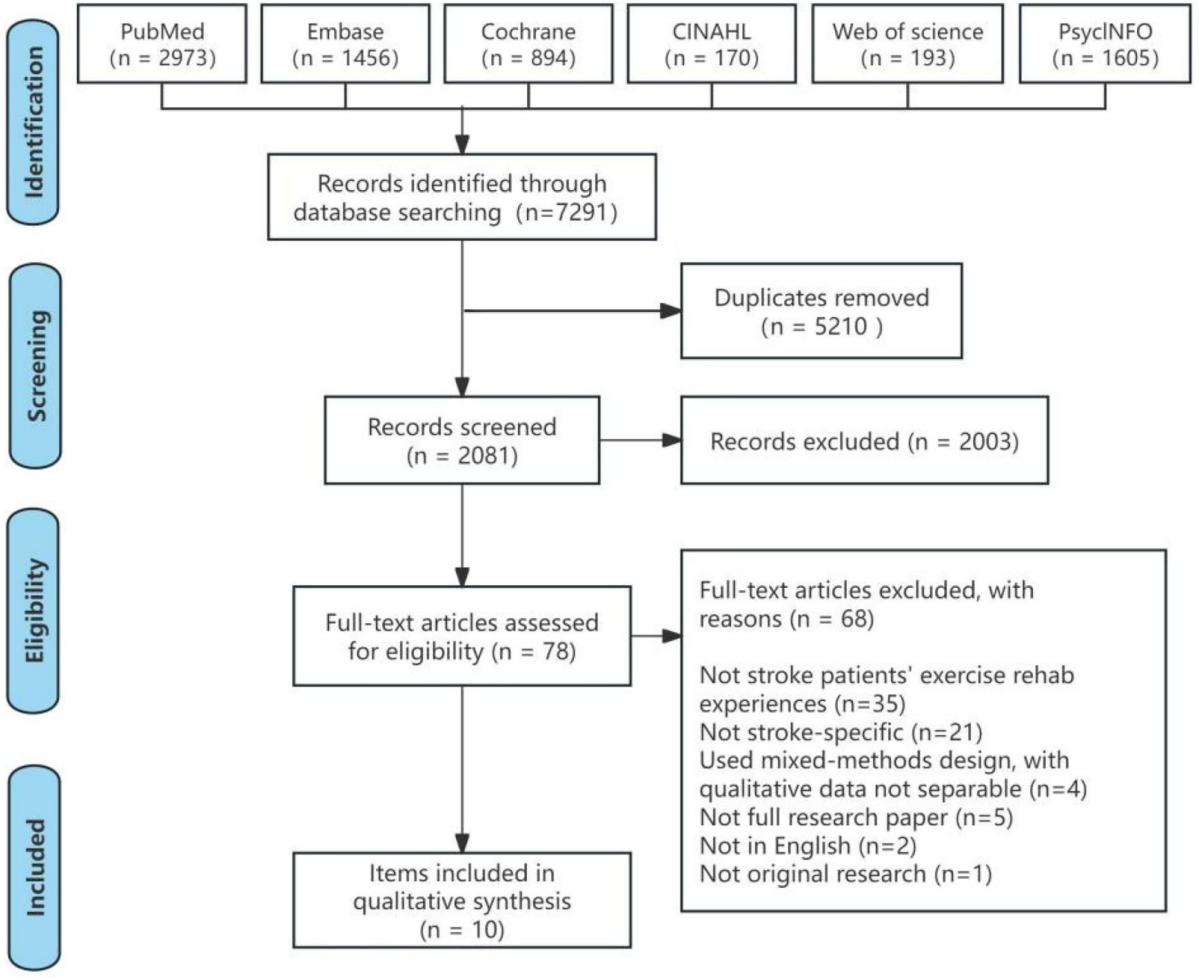
PRISMA flowchart.

As shown in Figure 1, the database search identified 7,291 studies. After duplicates were removed, 2,081 studies remained. Following title and abstract screening, 2,003 studies were excluded, leaving 78 for full-text evaluation. Ultimately, 10 studies (16, 33–41) satisfied the inclusion criteria.

### 3.2 Closed-Loop model

**Figure 2:**
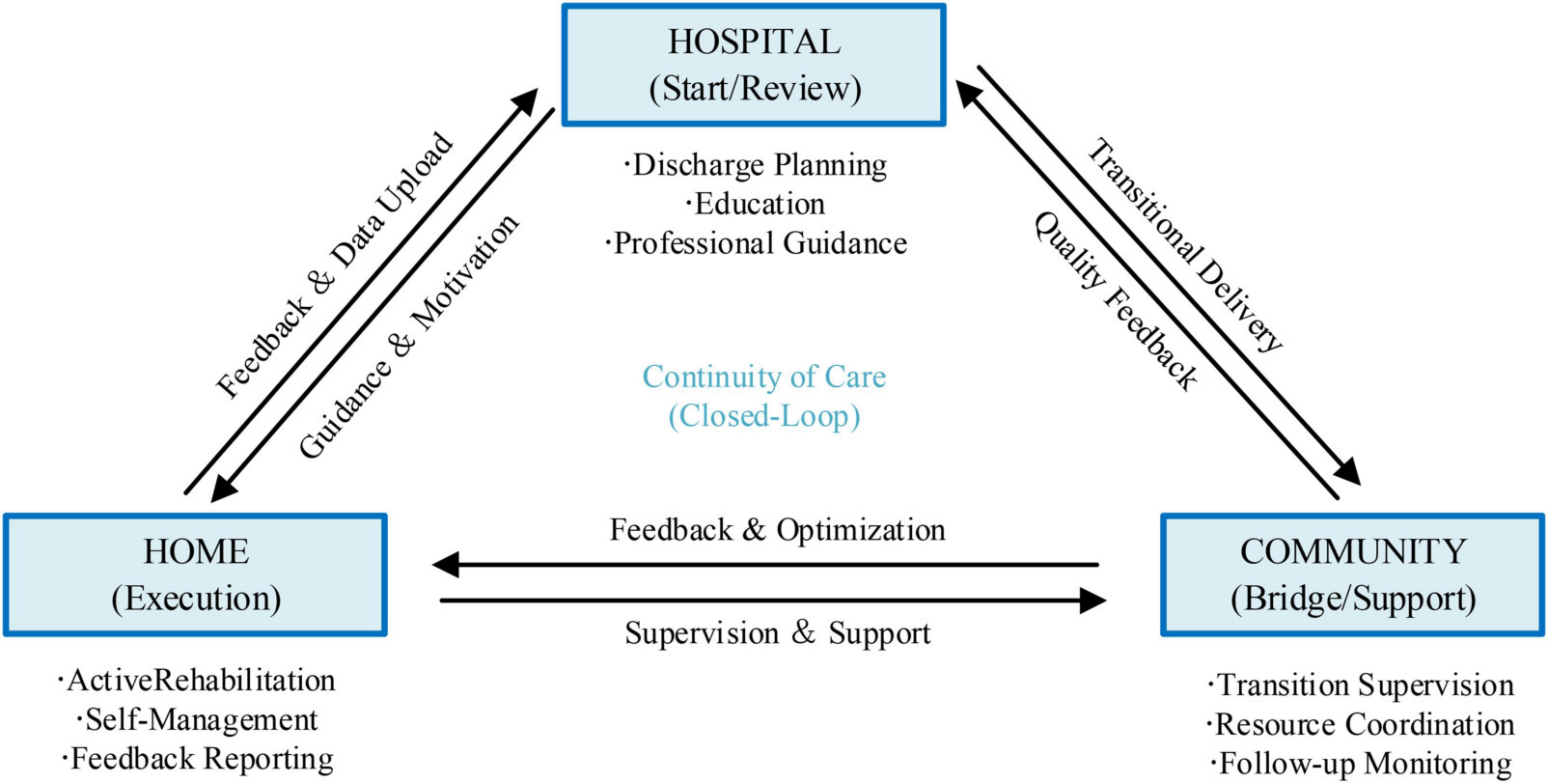
Closed-loop model of rehabilitation care.

The closed-loop model in this study illustrates the continuous interaction between the hospital, community, and home settings in stroke rehabilitation. As shown in Figure 2, the hospital provides initial guidance and discharge planning, which is followed by community support and, finally, home-based self-regulation and feedback. This cyclical process ensures continuous care, with feedback flowing between settings to maintain rehabilitation adherence. The model emphasizes the importance of continuity of care across these stages, facilitating sustained patient engagement and rehabilitation success.

### 3.3 Quality evaluation

The results of the quality assessment for the included studies are presented in Table 1. All studies demonstrated strong performance in methodological consistency (items 1–5), presentation of participant perspectives (item 8), ethical compliance (item 9), and evidence supporting conclusions (item 10), achieving 100% consistency (10/10). Research methods, data analysis, and interpretation of conclusions exhibited logical coherence; participant perspectives were fully articulated; and all studies received ethical approval.

**Table 1:** Results of quality appraised.

Included Studies	1	2	3	4	5	6	7	8	9	10
Khoshbakht Pishkhani et al. (33)	Y	Y	Y	Y	Y	U	U	Y	Y	Y
Pereira et al. (34)	Y	Y	Y	Y	Y	Y	Y	Y	Y	Y
Van Dongen et al. (35)	Y	Y	Y	Y	Y	U	U	Y	Y	Y
Yoshida et al. (36)	Y	Y	Y	Y	Y	U	Y	Y	Y	Y
Chau et al. (37)	Y	Y	Y	Y	Y	U	U	Y	Y	Y
Kelly et al. (38)	Y	Y	Y	Y	Y	Y	U	Y	Y	Y
Levy et al. (39)	Y	Y	Y	Y	Y	Y	Y	Y	Y	Y
Krawczyk et al. (40)	Y	Y	Y	Y	Y	Y	Y	Y	Y	Y
Smith et al. (41)	Y	Y	Y	Y	Y	Y	U	Y	Y	Y
Zhang et al. (16)	Y	Y	Y	Y	Y	U	U	Y	Y	Y

JBI Critical Appraisal Checklist for Qualitative Research. Items 1. Whether the philosophical perspective aligns with the research methods, Items 2. Whether the research methods correspond to the research questions or objectives. Items 3. Whether the research methods are consistent with the data collection approach. Items 4. Whether the research methods align with the data analysis and presentation approach; Items 5. Whether the research methods are consistent with the interpretation of findings; Items 6. Whether the researcher's concepts and values that may influence the study are clarified; Items 7. Whether the researcher's influence on the study and the study's influence on the researcher are articulated; Items 8. Whether participants’ itended meanings are fully represented. Items 9. Whether the study adheres to current ethical standards and possesses research ethics approval recognized by academic institutions, Items 10. Whether the study's conclusions are derived from data analysis and interpretation) Y = yes; U = unclear.

However, notable deficiencies were observed in the reflective research entries (Entries 6 and 7): Entry 6—whether the researcher' s perspectives and value positions that may influence the study were clarified—showed an uncertainty rate of 50% (5/10); Entry 7—description of the relationship between the researcher and participants and its impact on the study—had an uncertainty rate of 60% (6/10). These findings indicate that, while existing research provides robust empirical evidence on motivational maintenance and continuity of care in home-based rehabilitation, systematic reflection on the researcher's positionality remains limited.

### 3.4 Basic characteristics included in the study

Table 2 presents the basic characteristics of the 10 included qualitative studies, covering 263 stroke survivors from eight countries between 2018 and 2023. The mean age of participants was 63.2 years (±7.5), with 47.8% female. Most participants were in the subacute to chronic phase post-stroke, ranging from 2 months to 22 years after onset. The primary settings were home-based (60%) and community rehabilitation (40%) programs.

**Table 2:** Basic characteristics of included literature.

Included Studies	Country	Research Design	Scene Type	Nursing/Rehabilitation Program Participation	Sample size (n)	Average age (years)	Women (%)	Time since stroke onset (range)	Primary Analytical Methods	Research Focus and Core Themes
Khoshbakht Pishkhani et al. (33)	Iran	Qualitative Research	Community Rehabilitation Center	Nurse and speech therapist	20	67	33	0.5–2 months after stroke	Content Analysis	Identifying Factors Influencing Adherence: Patient Characteristics, Team Collaboration, and System Support
Pereira et al. (34)	Portugal	Qualitative Research	Home Rehabilitation	Nurses and physical therapists	24	67	50	1–6 months after stroke	Thematic Analysis	Revealing the patient’s journey to “regain control of life” and the tripartite collaborative mechanism
Van Dongen et al. (35)	Iceland/Netherlands	Qualitative Research	Home and Community Rehabilitation	Nurses and physical therapists	10	64	30	6–18 months after stroke	Thematic Analysis	Focus on the dynamic process of home-based exercise, adaptation, and family support
Yoshida et al. (36)	Japan	Qualitative Research	Community Rehabilitation	Rehabilitation Therapist	20	65.8	45	2–3 months after stroke	Thematic Analysis	Explore the origins and shifts in motivation to uncover patients’ support needs during transitional phases
Chau et al. (37)	Hong Kong, China	Qualitative Research	Home and Community Rehabilitation	Nurses and physical therapists	50	61.6	32	24–72 months after stroke	Thematic Analysis (Braun & Clarke Method)	Exploring the rehabilitation experiences and needs of community stroke survivors, and proposing a continuity of care strategy based on family and volunteer support
Kelly et al. (38)	Australia	Qualitative Research	Hospital and Home Rehabilitation	Nurses and physical therapists	84	55	50	3–12 months after stroke	Thematic Analysis	Exploring the rehabilitation transition experience of indigenous stroke patients from hospital to home, emphasizing family roles and cross-cultural nursing collaboration mechanisms
Levy et al. (39)	Australia	Semi-structured home interviews	Home Rehabilitation	Rehabilitation Therapist	20	63	40	6–18 months after stroke	Thematic Analysis (TDF/COM-B Framework)	Exploring Upper Limb Movement Compliance Disorders and Promotion Mechanisms
Krawczyk et al. (40)	Denmark	Focus Group Qualitative Research	Community Rehabilitation	Rehabilitation Therapist	35	69	40	1–3 months after stroke	Content Analysis	Analyze attitudes toward physical activity, barriers to participation, and preferences for group exercise
Smith et al. (41)	United Kingdom	Semi-structured interview	Community Rehabilitation	Rehabilitation Therapist	16	65	37	6–24 months after stroke	Reflexivity Theme Analysis	Exploring the effects of group exercise experiences, self-efficacy, and social support
Zhang et al. (16)	China	Phenomenological Qualitative Research	Home and Community Rehabilitation	Nurses and physical therapists	28	69	42	5–12 months after stroke	Colaizzi Analysis	Exploring Factors Influencing Home Exercise Adherence: Individual, Family, and Follow-Up Support

Methodologically, 80% of the studies employed thematic analysis to examine the intrinsic construction of meaning and behavioral motivation mechanisms in collective experiences. All studies underwent ethical review and received informed consent. Sample sizes ranged from 10 to 50 participants (mean 26.3), with most studies employing semi-structured interviews or focus group discussions to balance data depth and diversity.

Regarding research content, 90% of the literature (16, 33–37, 39–41) examined maintaining exercise motivation, self-efficacy, and belief reconstruction in patients within home environments. 80% (16, 34–38, 40, 41) highlighted the role of family and social support systems in promoting exercise adherence. 70% (16, 33–35, 37–39) explicitly emphasized the critical importance of nursing continuity, including community nurse follow-ups, rehabilitation guidance, and telephone supervision. Collectively, these studies indicate that, in home settings without hospital supervision, sustained support from healthcare providers and individual regulation of motivation are central elements for promoting rehabilitation adherence.

### 3.5 Meta-aggregation flowchart

**Figure 3:**
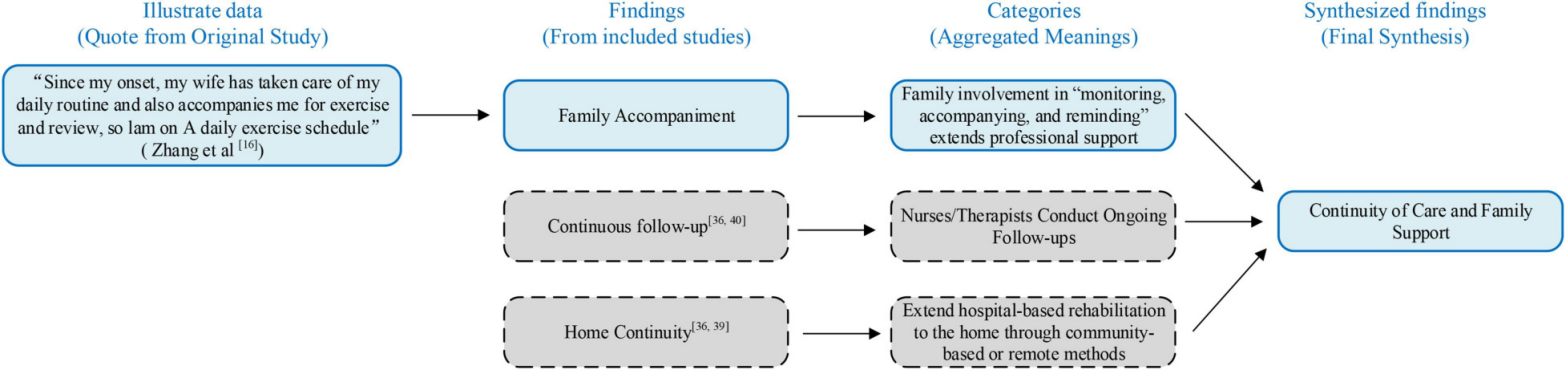
Meta-aggregation flowchart (JBI framework example).

The meta-aggregation process was used to synthesize findings from the included studies, as shown in Figure 3. This process involves transforming illustrative data (participant quotes) into findings, grouping similar findings into categories, and ultimately synthesizing them into synthesized findings. These steps ensure that data is systematically integrated into coherent, actionable insights. The flowchart provides a clear visual representation of how individual findings were categorized and synthesized to derive overarching conclusions for practice.

### 3.6 Key findings

Table 3 presents an overview of the comprehensive findings. Across 10 studies, 48 research findings were identified and categorized into 12 and four overarching themes. Among these findings, 36 were rated as clear and 12 as plausible.

**Table 3:** Specific topics and supporting introductions.

Findings	Categories	Synthesized findings
Family support (16, 37)	Insufficient follow-up guidance after discharge, with family-based resources struggling to take over.	Gaps in Nursing Guidance and Follow-Up During the Discharge Transition
Accessibility of rehabilitation services (40)		
Resource constraints (16)		
Insufficient support (37)		
Transportation issues (36, 40)	Transportation and accessibility constraints limit access to community-based rehabilitation services.	
Accessibility (36)		
Geographical barriers (16)		
Environmental adaptability (36, 40)		
Access barriers (41)		
Family involvement (16, 33, 37)	Include family members in discharge planning and establish a closed-loop follow-up system.	
Follow-up closure (16, 37)		
Systemic support (16, 33)		
Treatment adherence (40)		
Self-supervised learning (36, 39, 40)	Self-Directed Training and Goal Grading: Making Training a “Daily Habit”	Self-Regulation and Motivation Maintenance Mechanisms in Home Settings
Goal Setting (36, 40)		
Behavior Modification (39, 40)		
Continuous Training (36, 39)		
Experience Feedback (16, 33, 36)	Success/failure experiences and physical state jointly shape motivation.	
Physical Condition (16, 33)		
Psychological Support (36)		
Professional Feedback (39, 40)	Professional feedback and encouragement enhance self-efficacy and willingness to take on challenges.	
Self-Efficacy (36)		
Willingness to Take on Challenges (39, 40)		
Supportive Feedback (39)		
Peer support (36, 40, 41)	Companions/groups provide a sense of security and perseverance.	The Promoting Role of Family and Community Support
Group dynamics (36, 41)		
Social mutual aid (36, 41)		
Sense of security (36, 40)		
Professional supervision (36, 39, 40)	Prefer group exercise under professional supervision	
Group exercise (36, 40)		
Physical activity (36, 39)		
Professional guidance (40)		
Sense of participation (36)		
Emotional Support (16, 37)	Family Emotional and Logistical Dual Support	
Family Accompaniment (16, 37)		
Continuous follow-up (36, 40)	Nurses/Therapists Conduct Ongoing Follow-ups: Guidance—Monitoring—Feedback Form a Closed-Loop System	Continuity of Care and Family Support
Feedback mechanism (36)		
Supervision (36, 40)		
Treatment monitoring (40)		
Rehabilitation closed loop (36)		
Family Accompaniment (16, 37)	Family involvement in “monitoring, accompanying, and reminding” extends professional support.	
Family Involvement (37)		
Professional Continuity (16)		
Supervision and Accompaniment (16)		
Remote Support (36, 39, 40)	Extend hospital-based rehabilitation to the home through community-based or remote methods	
Community Rehabilitation (36, 40)		
Home Continuity (36, 39)		
Rehabilitation Extension (39, 40)		

#### 3.6.1 Nursing guidance and follow-up gaps during the discharge transition

During the rehabilitation phase, as stroke patients transition from hospital to home, research consistently highlights a critical issue: gaps in care guidance and insufficient follow-up. 90% of studies explicitly report that patients lack ongoing rehabilitation guidance and systematic nursing follow-up after discharge. This gap undermines adherence to and the safety of home-based exercise rehabilitation. Most patients experience uncertainty and isolation during the initial home transition, struggling to translate hospital-prescribed exercise regimens into actionable home-based plans (16, 38). Simultaneously, limited community rehabilitation resources and transportation barriers exacerbate transitional vulnerability (40), highlighting persistent structural gaps in care continuity between hospital and home settings.

Descriptive Theme 1: Insufficient continuous guidance after discharge leaves patients lacking direction for rehabilitation. Multiple studies indicate that discharge education often consists of a single briefing during the final days of hospitalization, without ongoing guidance extending into the home environment. Zhang et al. (16) documented in a Chinese study that 83% of patients received no phone calls or home visits from healthcare providers within one month of discharge, leaving them uncertain about safely performing home exercises. Similarly, Pereira et al. (34) indicated through interviews conducted one and six months after discharge that patients commonly reported a lack of a clear rehabilitation roadmap during the initial phase, relying solely on recollections of verbal instructions provided by nurses before discharge. This resulted in substantial variations in exercise implementation. Kelly et al. (38) further showed in an Australian Aboriginal sample that this disruption in education and follow-up not only affects physical recovery but also diminishes patients psychological sense of security regarding being cared for and attended to, constituting the first discontinuity in continuity of care.

Descriptive Theme 2: Poor Accessibility of Community Rehabilitation Services and Absence of Follow-up Systems. Accessibility challenges represent a key barrier to continuity of care. In a Danish focus group study (40), more than 70% of patients with mild stroke were unable to attend community rehabilitation programs regularly due to transportation difficulties or fatigue. Patients commonly expressed a desire for rehabilitation centers closer to home or for home-based guidance. Studies in China and Iran (16, 33) indicated that limited community rehabilitation resources and the lack of effective information-sharing mechanisms between nurses and therapists rendered follow-up visits largely ineffective. Similarly, Kelly et al. (38) reported that spatial distance to services and uneven resource distribution led to prolonged neglect of rehabilitation needs among indigenous stroke patients, highlighting how institutional distance undermines continuity of care after hospital discharge.

Descriptive Theme 3: Establishing a Coordinated Continuity Mechanism Among Home, Community, and Hospital.To address these gaps, several studies have proposed optimization strategies. Kelly et al. (38) recommended a nurse-led tiered follow-up system as central, combined with a joint program integrating remote follow-up and family involvement to bridge the disconnect between guidance, execution, and feedback. Pereira et al. (34) highlighted the bridging role of family caregivers during transitions, noting that incorporating family support into the follow-up system effectively enhances patient adherence and confidence. Studies in Australia and the UK (39) demonstrate that remote rehabilitation feedback and personalized prescription updates foster a sustained sense of being cared for, fostering higher levels of exercise adherence. In summary, restructuring nursing guidance and follow-up systems requires nurses as coordinating hubs to establish a three-dimensional collaborative network connecting families, communities, and hospitals, achieving accurate continuity of care.

#### 3.6.2 Self-regulation and motivation maintenance mechanisms in home settings

Among the 10 included studies, 90% (16, 33–37, 39–41) focused on self-regulation and mechanisms for maintaining motivation during the home rehabilitation phase for stroke patients. Research indicates that, as patients transition from hospital to home settings, exercise behavior shifts from professional supervision to reliance on personal willpower, self-efficacy, and social incentives. Adherence to home-based exercise tends to decline three to six months after discharge, particularly without ongoing guidance and positive feedback (16, 39). However, adherence to home-based exercise and associated rehabilitation outcomes show notable improvements when patients establish positive feedback loops through goal setting, self-monitoring, and achievement feedback (40). The core challenge lies in sustaining rehabilitation motivation in unsupervised home settings through self-efficacy, reinforcement of motivation, and feedback support.

Descriptive Theme 1: Self-Directed Training and Goal Progression: Establishing Exercise as a Daily Habit.Self-regulation in home-based rehabilitation begins with fostering goal awareness and integrating exercise into daily routines. In a Danish focus group study (40), all participants identified integrating exercise into daily routines as crucial for rehabilitation success. Approximately 68% of participants reported that substituting formal training with everyday activities, such as incorporating exercise into errands, stair climbing, or kitchen tasks, effectively addressed time constraints and limited training space. Japanese research (36) similarly noted that patients developing spontaneous behaviors, such as practicing in the hallway during the subacute phase, reflects a psychological shift from external to self-directed motivation. Multiple studies emphasize that nurses or therapists should assist patients in setting measurable, phased home goals during discharge education to structure the self-training process (34).

Descriptive Theme 2: Success and Failure Experiences and Physical Condition Jointly Influence Motivational Fluctuations. Motivational maintenance is not a linear process but a dynamic system jointly influenced by physical recovery, emotional experiences, and social interactions. Yoshida et al. (36) revealed that patients' exercise motivation often fluctuates between experiences of success, fatigue, and renewed motivation. For example, one respondent stated, “When my recovery is going well, I want to challenge myself with more demanding training, but when I encounter pain or misunderstanding from others, I lose confidence.” Research by Levy et al. (39) in Australia also shows that positive feedback and visible progress enhance motivation, whereas a sense of stagnation is a primary factor leading patients to discontinue rehabilitation. Data indicate that approximately 60% of patients increase their average daily training time by about 30% after receiving a single instance of successful feedback. Consequently, studies recommend incorporating feedback mechanisms, including nurse follow-ups, digital logging, or family observation, into home-based rehabilitation to reinforce patients' positive motivation loops.

Descriptive Theme 3: Professional Feedback and Social Motivation Enhance Self-Efficacy. Continuous feedback from nurses and rehabilitation therapists is a crucial external motivator during the home-based phase. Levy et al. (39) noted that periodic feedback sessions allow patients to observe the results of their efforts, motivate them, prompt professionals to adjust plans, and form an interactive incentive model. Van Dongen et al. (35) demonstrated that patients receiving telephone follow-ups or text reminders from nurses were more likely to adhere to training rather than postpone it, particularly when nurses provided specific guidance. Qualitative research in China (16) demonstrated that family members' praise, companionship, and reminders produced similar positive effects, with 70% of respondents reporting that feelings of being cared for and encouraged positively correlated with rehabilitation progress. Thus, professional feedback and social motivation constitute a dual-efficacy support system during home-based rehabilitation. This approach compensates for the lack of direct supervision while fostering patients' autonomous growth through emotional motivation.

#### 3.6.3 The promoting role of family and community support

80% of studies (16, 34–38, 40, 41) emphasize the essential role of family and community support systems in facilitating home-based rehabilitation. Research consistently indicates that social support is fundamental in sustaining patients' exercise adherence and emotional well-being, especially during the post-discharge period when professional supervision is unavailable. Family members, peer groups, and community rehabilitation networks collectively play important roles in emotional motivation, behavioral monitoring, and resource coordination (16, 38, 40). Approximately 72% of patients reported that encouragement and companionship from family members served as the main drivers for sustained rehabilitation. Community exercise groups organized by nurses or rehabilitation therapists strengthened social connections and recovery confidence (41). This evidence suggests that a dual family-community support system constitutes the essential foundation for implementing continuity of care within the home setting.

Descriptive Theme 1: Peer and Group Support Promote Emotional Connection and Psychological Security. Qualitative studies across various countries demonstrate that peer and group support substantially enhances patients' rehabilitation motivation and sense of social belonging. A Danish focus group study (40) found that 85% of patients with mild stroke preferred group exercise, citing security and companionship as the primary benefits of exercising with others. In Yoshida et al. 's Japanese study (36), patients reported that interactions and mutual encouragement with fellow participants alleviated loneliness, while the shared rehabilitation environment reinforced emotional stability and persistence toward goals. Smith et al. (41) further indicated that community exercise groups provide physical training opportunities and foster peer accountability, enabling participants to sustain self-discipline through mutual reminders and shared achievements. These findings indicate that peer relationships are valued in emotional support and self-identity, as essential psychological regulatory mechanisms during home-based rehabilitation.

Descriptive Theme 2: Professional Supervision Promotes Trust and Engagement Motivation. Beyond emotional connection, professional supervision is identified in multiple studies as a critical factor in ensuring exercise safety and promoting engagement. In Krawczyk et al. 's (40) study, respondents consistently preferred exercising under professional supervision, particularly during the early stages when confidence in managing exercise risks and recovery pacing was limited. Smith et al. (41) indicated that professional guidance instills a sense of being acknowledged and validated within the rehabilitation process. Patients ‘ trust and engagement levels increased substantially when nurses or physical therapists explained the principles underlying movements and adjusted training plans. Chua et al. (37) found that regular remote guidance and feedback videos fostered patients' sense of being attended to, with this perceived social presence effectively reducing dropout rates. Professional supervision addresses technical guidance requirements while enhancing rehabilitation motivation by promoting psychological security.

Descriptive Theme 3: Family Emotional and Practical Support Enhances Rehabilitation Adherence. Family support is widely recognized as a fundamental prerequisite for home-based rehabilitation. Zhang et al. (16) confirmed that 92% of patients considered family companionship and encouragement the primary motivators in rehabilitation. Typical expressions included statements such as “My wife reminds me to exercise every day, and I must show her my progress.” Yoshida et al. (36) also observed that patients often maintained training motivated by a desire not to disappoint their families. An Iranian study (33) added a cultural perspective, highlighting that families provide emotional support and practical assistance, including transportation and daily care, allowing patients to focus on rehabilitation exercises. Pereira et al. (34) established that patient adherence increased by an average of 27% when family members participated in nurse follow-ups or remote check-ins. These findings suggest that emotional support and practical assistance from family members jointly constitute a dual internal-external driving force for sustained rehabilitation behavior, serving as a critical component for ensuring continuity of care at the family level.

#### 3.6.4 Continuity of care and family support

70% of studies (16, 33–35, 37–39) explicitly indicate that nurse-led follow-up integrated with family collaboration constitutes the primary pathway for sustaining continuity in home-based rehabilitation (34, 38, 39). Collectively, these studies indicate that after patients leave hospital supervision, implementing a closed-loop support system through nursing follow-ups, either in-person or remote, together with community-linked family involvement that facilitates informational and emotional exchange, substantially enhances exercise adherence and psychological security. Conversely, in the absence of continuous nurse-patient interaction or when family care is disconnected from professional guidance, patients' rehabilitation motivation and adherence decline rapidly (16). This theme emphasizes that nursing continuity is not an isolated action but a cross-context system of coordinated collaboration.

Descriptive Theme 1: Nurse-Led Follow-Up and Feedback Establishing a Rehabilitation Closed Loop. Multiple studies indicate that nurses function as information providers during the post-discharge phase and as the central coordinators for rehabilitation monitoring and emotional support. Pereira et al. (34) documented in longitudinal interviews in Portugal that patients receiving nurse follow-up via telephone or home visits at one and six months post-discharge exhibited approximately 30% higher adherence to home exercises and substantially improved control over their recovery pace. Australian research (39) further confirmed the positive feedback loop: feedback enabled patients to recognize that nurses' guidance directly contributed to progress, enhancing confidence in maintaining training. Similarly, a Hong Kong study (37) indicated that a hybrid approach combining remote video follow-ups with telephone call-backs enhanced adherence and reinforced the psychological experience of feeling attended to. Thus, nurse-led periodic follow-ups provide structural support for rehabilitation, establishing a dynamic closed-loop of guidance, execution, and feedback.

Descriptive Theme 2: Family Members Reinforce Professional Guidance Through Supervision and Companionship. Family members fulfill dual roles as supervisors and motivators within the continuity of care framework. Zhang et al. (16) illustrated that a multi-perspective study in China reported that patients with family involvement in follow-up care performed daily exercises approximately 1.4 times more frequently than those without family support. Through observation, reminders, and documentation of rehabilitation progress, family members reinforce the reach of nursing guidance within the home environment. Pishkhani et al. (33) indicated that Similarly, within Iran's home-care context, wives and daughters act as implementation assistants for patients' rehabilitation plans, supervising daily exercises and providing encouragement based on guidance from nurses. Moreover, Yoshida et al. (36) reported in a Japanese sample that patients frequently maintained exercises to avoid disappointing family members. This form of family involvement, integrating emotional responsibility with supervision of practice, effectively supplements the intermittent nature of professional follow-ups, ensuring the sustainability and practicality of rehabilitation guidance.

Descriptive Theme 3: Strengthening Continuity of Care Through Community and Hospital Collaborative Networks. Continuity of care extends beyond nurse-family interactions and depends on systematic collaboration between community services and hospitals. Kelly et al. (38) reported in a study of Australian Aboriginal populations that multi-level community and hospital collaborative networks, including nurses, therapists, and community health workers, effectively prevent patients from becoming unreachable after discharge. Levy et al. (39) further recommended to develop cross-sector information platforms to allow community nurses to access real-time updates on patients' rehabilitation progress and prescription adjustments, thereby ensuring consistent care across institutions. Van Dongen et al. (35) highlighted a psychological aspect: patients are more likely to continue training at home rather than relearn exercises when they perceive continuity between hospital care and community support. Thus, constructing a nurse-centered collaborative network linking hospital resources with community services represents a key strategy for ensuring genuine continuity of care.

### 3.7 Dependability and credibility

Table 4 presents the ConQual summary of the synthesized findings. All ten articles included in this study satisfied the core criteria of the JBI qualitative research quality assessment, including methodological consistency, ethical compliance, and evidence supporting conclusions; therefore, all were retained. For the dependability rating, we followed the method described in Section 2.8.1 by evaluating Items 2, 3, 4, 6, and 7 of the JBI Critical Appraisal Checklist for Qualitative Research. As shown in Table 1, Six studies (34, 36, 38–41) achieved 4–5 “Yes” ratings and received no downgrade, whereas four studies (16, 33, 35, 37) achieved 3 “Yes” ratings and were downgraded by one level, mainly due to unclear reporting on Items 6–7 (reflexivity-related items). Accordingly, the dependability of the synthesized findings was downgraded by one level. Regarding credibility, the synthesized findings were supported by unequivocal evidence, consisting of direct participant quotes and clear illustrations from the original studies that established a logical link between the data and the findings. Therefore, no downgrade was applied to credibility. Consequently, all four synthesized findings received an overall ConQual rating of moderate, which should be considered when interpreting the strength of practice implications.

**Table 4:** Conqual summary of the findings.

Synthesized findings	Type of research	Dependability	Credibility	ConQual score
Gaps in Nursing Guidance and Follow-Up During the Discharge Transition	Qualitative	Downgrade 1 level	No downgrade	Moderate
Self-Regulation and Motivation Maintenance Mechanisms in Home Settings	Qualitative	Downgrade 1 level	No downgrade	Moderate
The Promoting Role of Family and Community Support	Qualitative	Downgrade 1 level	No downgrade	Moderate
Continuity of Care and Family Support	Qualitative	Downgrade 1 level	No downgrade	Moderate

ConQual ratings were determined by downgrading for dependability (Section 2.8.1) and credibility (Section 2.8.2).

## 4 Discussion

### 4.1 Gaps in nursing guidance and follow-up during the discharge transition

The transition from hospital care is not a seamless continuation of the medical pathway but a high-risk period of discontinuity. Our findings reveal three forms of discontinuity post-discharge: information, relational, and resource gaps. First, the information gaps identified in this study highlight the pervasive challenges in translating clinical guidelines into practice. Although the European Stroke Organisation (ESO) (42) explicitly recommends providing standardized rehabilitation education and personalized prescriptions before discharge, our synthesis indicates that static education is insufficient to address the complex challenges of the home environment. This qualitative finding complements objective data, observing a rapid decline in adherence among stroke patients (43). Our qualitative evidence further elucidates the underlying psychological mechanism. This decline stems not only from functional limitations but also from safety-related anxiety caused by a lack of real-time guidance. This suggests that existing health education models fail to fill the behavioral adaptation void during the transition from a controlled hospital setting to a complex home environment.

Second, the relational gaps emphasized in this study reflect significant differences between stroke rehabilitation and other chronic disease management models. Unlike the well-established home-based exercise models in cardiac rehabilitation (44), stroke rehabilitation exhibits a higher degree of technical dependence and a critical need for real-time feedback (45). In the absence of continuous follow-up by specialist nurses or physical therapists, stroke survivors may develop learned helplessness and abandon their training programs due to a lack of immediate correction and positive reinforcement (46). This reflects a rupture in professional support during the transition home. The inadequacy of existing primary healthcare systems in providing high-intensity, specialized follow-up remains a systemic cause of suboptimal long-term exercise adherence.

Third, resource gaps expose the global challenge of inequitable medical service distribution. In centralized systems such as China, discontinuity often stems from the concentration of high-quality rehabilitation resources in tertiary hospitals, leaving primary care facilities ill-equipped to provide specialized follow-up (47). Conversely, even in decentralized systems like Denmark, where community-based services are more robust, patients still encounter transition fatigue due to fragmented communication between municipal providers and acute care sectors (48). In the context of this study, this implies that rehabilitation adherence is no longer merely a matter of individual motivation but an ecological issue constrained by the accessibility of systemic resources.

In summary, the discharge transition is a phased, dynamic process involving behavioral adaptation, reconstruction, and integration (16). Based on a study of community stroke patients in Hong Kong, Chau et al. (37) proposed a rhythmic continuity of care model encompassing pre-discharge preparation, discharge-day handover, and post-discharge follow-up, which provides a practical reference for this study. Building on this, this study further proposes that transitional care should be temporally continuous and experientially continuous. Consequently, future discharge support should shift from education to behavioral support, achieving this transformation through remote follow-ups, hybrid care pathways, and home-adaptive technologies.

Beyond systemic gaps, home-based rehabilitation relies on the transition from external compliance to autonomous self-regulation, a process aligned with the COM-B model (49). In the Capability and Opportunity dimensions, our findings suggest that environmental constraints and psychological readiness are barriers to adherence. This qualitative insight complements quantitative data (50) showing that environmental factors significantly predict post-stroke exercise persistence. Unlike studies focusing solely on movement techniques (41), we emphasize life-embedded exercise to alleviate the psychological burden, treating the home environment as an active affordance for recovery. Regarding motivation, home-based training lacks immediate feedback, making patients more susceptible to frustration, pain, and emotional fluctuations (39). This explains the quantitative trend (45) of declining adherence post-discharge. To rebuild the psychological scaffolding, we propose using objective feedback metrics [e.g., Borg RPE or wearable data (51)] to provide the immediate reinforcement previously supplied by clinicians. Therefore, a closed-loop strategy that integrates goal stratification, real-time feedback, and problem-solving follow-ups is essential.

### 4.2 The role of family and community support in enhancing rehabilitation sustainability

When rehabilitation transitions from institutional settings to the home environment, motor rehabilitation for stroke patients evolves from an individual health behavior to a social practice influenced by family structures, social relationships, and community resource s (35). The integrated findings of this study indicate that a three-tiered support network—comprising family, peers, and community—is essential for patients' sustained engagement. Through three interlinked mechanisms, the network reinforces adherence to rehabilitation. Emotional support acts as the primary driver of rehabilitation motivation. While quantitative evidence frequently highlights the strong correlation between family companionship and recovery outcomes (52, 53), our qualitative synthesis further elucidates the underlying mechanism: family affirmation does not merely provide encouragement but helps patients reconstruct their identity and alleviate the sense of illness and self-doubt. This emotional buffering is particularly critical in stroke recovery to counter the psychological stress of solitary training. Unlike cardiac rehabilitation (54) that emphasizes functional monitoring, stroke rehabilitation requires a higher degree of relational continuity to maintain the patient's intrinsic drive.

Additionally, Structural support provides the practical conditions necessary for sustained rehabilitation implementation. Community-level resources and peer connections trigger long-term psychosocial effects through peer connections, fostering role identification and cohesion. This aligns with quantitative findings suggesting that group-based interventions significantly enhance self-efficacy compared to isolated home exercise (55). Through mutual reminders and shared achievements, patients undergo an identity transformation from passive care-receivers to active rehabilitators (56), which ensures adherence even after professional supervision is withdrawn. Therefore, nursing practice should evolve from singular patient education to supporting the construction of a comprehensive ecosystem. Nurses should instruct patients in training techniques and design supportive structures, such as integrating family members as rehabilitation collaborators and facilitating community peer support networks (36). This semi-supervised, semi-social participation model expands the accessibility of rehabilitation services, transforming recovery from an isolated task into a life practice embedded within relational and management continuity, thereby enhancing the sustainability of interventions.

### 4.3 Continuity of care and family support

Effective home-based exercise rehabilitation is unlikely to be achieved through a single intervention alone; rather, it requires establishing a guidance–monitoring–motivation closed-loop system coordinated by nurses and implemented collaboratively across hospitals, communities, and homes.

During the guidance phase, nurses must transition from traditional educators to rehabilitation facilitators. This process necessitates thoroughly considering the patient's home environment and caregiving resources to prevent the disconnect when hospital prescriptions are directly applied to home settings (37). This aligns with recent stroke rehabilitation guidelines (42), which emphasize that personalized, goal-oriented prescriptions are more effective than generic education in preventing the post-discharge service gap.

Regarding monitoring, our integrated findings suggest that minimal datasets of key metrics are crucial for sustaining long-term adherence (41). Specific indicators may include daily training steps/duration, Borg-RPE fatigue scores, etc (51). These metrics are easily understood by patients and families while effectively reflecting training efficacy. Nurses can maintain continuity in rehabilitation services through remote or in-home follow-ups at key time points. Consistent with quantitative evidence on telerehabilitation (57), scheduled remote follow-ups provide a rhythmic structure that reduces patient dropout rates by offering professional presence even in the absence of face-to-face contact.

Regarding motivation, this study highlights that stroke patients' rehabilitation adherence is driven more by value-based factors than by purely medical rationality (39, 40). Therefore, a key strategy is proactively establishing explicit connections between training and life's meaning. For example, interviews can guide patients in recalling family roles they wish to resume post-recovery, linking training to personal value goals (58). Additionally, group-based incentives, such as peer support groups and rehabilitation experience-sharing sessions, can strengthen patients' recovery identity and social reinforcement effects (59).

The Guidance-Monitoring-Motivation model constitutes a replicable, scalable, continuous care pathway framework. This includes nurses developing personalized home rehabilitation prescriptions before discharge, implementing tiered follow-ups, establishing peer support networks, and using patient training adherence and functional improvement as quality feedback indicators for continuous program optimization. Future research should employ implementation science and mixed methods to evaluate this model's effectiveness, scalability, and equity across diverse settings, thus advancing the practice translation of evidence-based care models.

### 4.4 Practical recommendations for clinicians

To effectively translate these findings into practice, it is essential to clarify the multidisciplinary synergy between nursing and physical therapy (PT). While stroke rehabilitation in many health systems is primarily led by PTs, who specialize in assessing movement impairments and prescribing task-specific training, our proposed nurse-centered closed-loop model should be interpreted as a coordination framework rather than a replacement. The primary distinction lies in their functional focus: PTs drive the clinical progression of exercise, whereas nurses complement this by addressing the continuity of care. Specifically, nurses bridge the discharge transition by monitoring adherence and safety, facilitating goal reviews, and connecting families to community resources—functions that patients in this synthesis identified as critical gaps.

Guided by this multidisciplinary framework, we propose the following considerations for optimizing home-based exercise rehabilitation. To bridge the care gap identified during the discharge transition, clinicians should prioritize an intensified follow-up schedule, particularly within the first two weeks post-discharge, followed by periodic reassessments at approximately one, three, and six months to address the fluctuating motivation observed in stroke survivors. To provide the professional supervision that patients reported as lacking, multimodal remote feedback, such as telephone calls, text messages, or remote monitoring sessions, should be employed to offer specific guidance on technique and safety. Furthermore, to bolster patient self-efficacy, clinicians should adopt a collaborative goal-setting approach, co-producing achievable short-term goals and reviewing progress through simple indicators (e.g., perceived exertion or step counts). Finally, given the vital role of social support networks, practitioners should explicitly define caregiver roles and facilitate stronger information handover between hospitals and community-based services to ensure that professional support and PT expertise are effectively extended into the home environment.

### 4.5 Limitations

This study, a meta-synthesis grounded in qualitative research, provides a comprehensive summary of stroke patients' experiences with home-based exercise rehabilitation from the perspective of nursing continuity. However, several limitations require objective consideration. First, the literature demonstrates considerable heterogeneity. The ten included studies spanned eight countries, revealing significant variations in healthcare systems and home care cultures across regions. Although methodological consistency was maintained through category consolidation and thematic extraction during synthesis, these contextual differences may still restrict the generalizability of the findings. Second, the qualitative reports varied in detail. Some studies inadequately described the researchers' data saturation process, making it challenging to assess their methodological rigor fully. Third, this study exclusively included English-language literature, potentially overlooking regional studies in other languages, particularly evidence regarding nursing practices in Latin America and Eastern Europe. Furthermore, the nature of meta-synthesized qualitative research precludes the quantitative measurement of intervention effects, allowing only experiential-level inferences about impact mechanisms. Future studies could integrate mixed-methods approaches and longitudinal tracking to validate the practical efficacy of continuity-of-care interventions in enhancing adherence and functional recovery.

## 5 Conclusion

This study systematically integrated ten qualitative studies from the perspective of nursing continuity, highlighting the potential importance of nursing support in stroke patients' experiences of home-based exercise rehabilitation. The synthesized findings suggest that perceived gaps in nursing continuity, individual self-regulation processes, and family-community social support networks may influence the home-based exercise rehabilitation experiences of stroke patients. These factors also point to potential challenges in current rehabilitation services, including transitional discontinuity in hospital-to-home care and limited adaptability across different rehabilitation phases. Drawing on these qualitative findings, we suggest that incorporating nursing continuity into the comprehensive management of stroke rehabilitation could be considered. This may be achieved through nurse-led, tiered follow-ups, family-collaborative personalized planning, and community linkage mechanisms to establish continuous, individualized feedback-driven care pathways. Furthermore, strengthening the role of community health services in rehabilitation follow-up and rehabilitation-related data collection and feedback support may help optimize the three-tiered collaborative rehabilitation network linking hospitals, communities, and families. Overall, this synthesis may provide evidence-informed insights and practical considerations for optimizing home-based exercise rehabilitation interventions in stroke patients.

## Data Availability

The data analyzed in this study is subject to the following licenses/restrictions: Datasets are derived from previously published studies, with restrictions (e.g., ethical approval, copyright) determined by the original research. No datasets are included in this manuscript or supplementary material. Requests to access these datasets should be directed to lilycmu@163.com.
