# Supplementary material for: Meta-synthesis of qualitative studies on home-based exercise rehabilitation experiences among stroke patients: a continuity of care perspective
Source: Front Rehabil Sci. 2026 Mar 4;7:1742902. doi: 10.3389/fresc.2026.1742902 (PMC12995860; doi:10.3389/fresc.2026.1742902)
Supplement: Supplementary file 1 [file Datasheet1.docx]

ENTREQ Reporting Checklist

|  | Item | Description | Page/LineNumber |
| --- | --- | --- | --- |
| 1 | Purpose of the synthesis | Research Question (derived from the study’s aim and scope): What are the key facilitators and barriers influencing stroke patients’ home-based exercise rehabilitation experiences, and how do nursing, family, and community support systems synergize to shape these experiences from a care continuity perspective?  Purpose: To address this question, the synthesis integrates qualitative evidence to clarify these factors and mechanisms, bridge the gap of fragmented findings, and inform the optimization of hospital-community-home collaborative care frameworks. | 1. Introduction |
| 2 | Rationale for synthesis methodology | The JBI meta-aggregation approach was selected because it is designed to synthesize qualitative findings into a format that is directly applicable to clinical practice and policy development, focusing on identifying commonalities across diverse studies. | 2.7 Data Synthesis |
| 3 | Protocol and registration | The study protocol was registered in PROSPERO. The registration number will be provided upon manuscript acceptance to comply with double-blind review requirements. No protocol amendments were reported. | 2.1 Research Design |
| 4 | Synthesis methodology detailed description | Adopted a three-step thematic synthesis process: 1. Extracted qualitative findings + open coding; 2. Grouped codes into subcategories; 3. Integrated subcategories into overarching themes. | 2.7 Data Synthesis |
| 5 | Inclusion criteria | 1. Study subjects: Patients with a confirmed diagnosis of ischemic or hemorrhagic stroke, encompassing the acute, recovery, and sequelae phases. 2. Phenomenon of Interest: Stroke patients’ subjective experiences during exercise rehabilitation, including key elements such as physical and psychological sensations during training, motivation for adherence, barriers and challenges, needs and expectations, and perceived outcomes. 3. Context: Exercise rehabilitation conducted in authentic home or community settings, including stroke units, rehabilitation institutions, community rehabilitation centers, outpatient clinics, and home environments. 4. Research Design: Original studies employing explicit qualitative methods, including phenomenology, grounded theory, ethnography, or thematic analysis, and mixed-methods studies from which qualitative data can be independently extracted. | 2.3 Inclusion and Exclusion Criteria |
| 6 | Exclusion criteria | Non-English literature; studies with inaccessible full texts, incomplete data, or missing critical information; mixed-method studies in which qualitative content is highly confounded with quantitative data and cannot be extracted separately; and duplicate publications. | 2.3 Inclusion and Exclusion Criteria |
| 7 | Information source | Databases: PubMed, Cochrane Library, Embase, CINAHL (EBSCO), Web of Science, and PsycINFO (EBSCO). | 2.2 Search Strategy  Supplementary File 1, Table S1 |
| 8 | Search strategy | Three-step search: 1) Initial (PubMed/CINAHL, subject terms + free-text); 2) Comprehensive (PubMed, Cochrane, Embase, etc., concepts: (stroke) AND (rehabilitation OR exercise) AND (qualitative OR interview)); 3) Snowball (reference lists). Period: database inception to Oct 19, 2025. | 2.2 Search Strategy |
| 9 | Study selection process | 1. De-duplication: EndNote (duplicates removed); 2. Two reviewers (title/abstract → full-text); 3. Disagreements resolved by third reviewer. | 2.4 Literature Screening and Data Extraction  Figure 1 PRISMA Flowchart |
| 10 | Study selection results | Initial: 8,562 → After deduplication: 7,317 → Title/abstract: 330 → Full-text: 10 included. | 3.1 Literature Search Results |
| 11 | Characteristics of included studies | 10 studies (2019–2023, 8 countries); n=187 (6–32/study); Data collection: Interviews (n=8), focus groups (n=2); Analysis: Thematic (n=6), grounded theory (n=3), phenomenology (n=1). | 3.6 Key Findings  Table 2 Basic Characteristics of Included Literature |
| 12 | Rationale for appraisal | To ensure methodological rigor of included studies, reliability of synthesized findings, and minimize bias from low-quality research, the JBI Critical Appraisal Checklist was employed. | 2.5 Quality Evaluation |
| 13 | Appraisal tool | JBI Critical Appraisal Checklist for Qualitative Research. The checklist comprises 10 items, each rated as Yes (Y), No (N), or Unclear (U). Studies were included in the meta-synthesis if six or more of the 10 criteria were rated Yes. | 2.5 Quality Evaluation |
| 14 | Appraisal process | Two reviewers appraised studies; disagreements resolved by third reviewer. No inter-rater reliability (Kappa) reported. | 2.7 Data Synthesis |
| 15 | Appraisal results | All included studies demonstrated strong performance in methodological consistency (items 1–5), presentation of participant perspectives (item 8), ethical compliance (item 9), and evidence supporting conclusions (item 10) (100% consistency, 10/10). Notable deficiencies were observed in reflective research entries: Item 6 (uncertainty rate 50%, 5/10) and Item 7 (uncertainty rate 60%, 6/10). | 3.7 Dependability and Credibility  Table 1 Results of quality appraised |
| 16 | Data extraction process | Two researchers independently extracted data via JBI-based tool, added stroke rehab-specific dimensions, resolved discrepancies with a third researcher. Conducted open coding on qualitative findings, extracted study info, sample, methodology, and core themes. | 2.6 Data extraction |
| 17 | Software used | Meta-synthesis: JBI SUMARI systematic review and meta-analysis tool. | 2.7 Data Synthesis |
| 18 | Number of reviewers involved in synthesis | Two reviewers involved in synthesis; discrepancies resolved by a third reviewer. | 2.7 Data Synthesis |
| 19 | Coding process | Open coding (inductive from raw findings) → axial coding (grouping codes into subcategories) → selective coding (integrating subcategories into themes). | 3.5 Meta-aggregation Flowchart |
| 20 | Comparison between studies | Compared findings across 10 included studies (covering 8 countries) to identify commonalities (e.g., post-discharge guidance gaps, low adherence drivers) and contextual differences (e.g., community resource accessibility variations). Assessed methodological consistency via JBI checklist and resolved discrepancies in findings through third-researcher review. | 3.4 Basic characteristics included in the study  Table 2 Basic Characteristics of Included Literature |
| 21 | Origin of themes | Themes (synthesized findings) were derived through a three-level meta-aggregation process: primary findings were extracted from included studies, grouped into categories based on conceptual similarity, and finally integrated into overarching synthesized findings. | 2.7 Data Synthesis  Figure 3 Meta-aggregation Flowchart |
| 22 | Illustration of themes with citations | Each synthesized theme was supported by direct citations from included studies. For example: 1) "Discharge guidance gaps" illustrated by Zhang et al. (2023, 83% patients had no post-discharge follow-up) and Pereira et al. (2021, lack of rehabilitation roadmap); 2) "Family support role" supported by Yoshida et al. (2021, family encouragement reduced dropout) and Khoshbakht Pishkhani et al. (2019, family practical assistance improved adherence). | 3.6 Key Finding  Table 3 Specific Topics and Supporting Introductions |
| 23 | Limitations of the synthesis | 1. Literature heterogeneity (8 countries, varied healthcare systems/cultures, generalizability restriction); 2. Varied qualitative report details (inadequate data saturation description); 3. Language bias (English-only, missing non-English regional studies); 4. Methodological limitation (no quantitative intervention effect measurement, only experiential inference). | 6 Limitations |
