# Supplementary material for: Meta-synthesis of qualitative studies on home-based exercise rehabilitation experiences among stroke patients: a continuity of care perspective
Source: Front Rehabil Sci. 2026 Mar 4;7:1742902. doi: 10.3389/fresc.2026.1742902 (PMC12995860; doi:10.3389/fresc.2026.1742902)
Supplement: Supplementary file 2 [file Table1.pdf]

Supplementary file 1

Table S1 **Search strategy**

Database	Indexed and keyword terms	N
PubMed	#1: (((((((((((stroke[MeSH Terms]) OR (stroke[Title/Abstract])) OR (post-stroke[Title/Abstract])) OR (apoplexy*[Title/Abstract])) OR (cerebrovascular accident[Title/Abstract])) OR (CVA[Title/Abstract])) OR (brain vascular accident[Title/Abstract])) OR (cerebral infarction[Title/Abstract])) OR (cerebral ischemia[Title/Abstract])) OR (cerebral hemorrhage[Title/Abstract])) OR (cerebrovascular disease[Title/Abstract])) OR (cerebral embolism[Title/Abstract])) OR (cerebral thrombosis[Title/Abstract])<br>#2: (((((((((((((((exercise[MeSH Terms]) OR (Rehabilitation[MeSH Terms])) OR (Exercis*[Title/Abstract])) OR (physical training[Title/Abstract])) OR (physical activit*[Title/Abstract])) OR (physical fitness[Title/Abstract])) OR (intervention*[Title/Abstract])) OR (rehabilit*[Title/Abstract])) OR (endurance*[Title/Abstract])) OR (resistance*[Title/Abstract])) OR (Functional Training[Title/Abstract])) OR (muscle training[Title/Abstract])) OR (aerobic*[Title/Abstract])) OR (gait training[Title/Abstract])) OR (fitness training[Title/Abstract])) OR (conditioning training[Title/Abstract])) OR (core training[Title/Abstract])<br>#3: ((((((((((Attitude[MeSH Terms]) OR (Perception[MeSH Terms])) OR (Attitude*[Title/Abstract])) OR (opinion*[Title/Abstract])) OR (Perception*[Title/Abstract])) OR (perspective[Title/Abstract])) OR (view*[Title/Abstract])) OR (Belief*[Title/Abstract])) OR (needs[Title/Abstract])) OR (feeling*[Title/Abstract])) OR (experience*[Title/Abstract])<br>#4: ((((((((((((((Interviews[MeSH Terms]) OR (Focus Groups[MeSH Terms])) OR (Grounded Theory[MeSH Terms])) OR (Qualitative Research[MeSH Terms])) OR (Narration[MeSH Terms]))) OR (Interview*[Title/Abstract])) OR (focus group*[Title/Abstract])) OR (grounded theory[Title/Abstract])) OR (qualitative research*[Title/Abstract])) OR (qualitative stud*[Title/Abstract])) OR (phenomenolog*[Title/Abstract])) OR (Narrati*[Title/Abstract])) OR (thematic*[Title/Abstract])) OR (ethnogra*[Title/Abstract])) OR (ethnolog*[Title/Abstract])<br>#1 AND #2 AND #3 AND #4	2973
Date of Search	*1977 to October 19, 2025*	
Embase (from Ovid)	#1: ('stroke'/exp OR 'post-stroke':ti,ab OR 'brain vascular accident':ti,ab OR 'cerebral infarction':ti,ab OR 'cerebral ischemia':ti,ab OR 'cerebral hemorrhage':ti,ab OR 'cerebrovascular disease':ti,ab OR 'cerebral embolism':ti,ab OR 'cerebral thrombosis':ti,ab OR 'cerebrovascular accident':ti,ab OR 'CVA':ti,ab)<br>#2: ('exercise'/exp OR 'gait training':ti,ab OR 'aerobic training':ti,ab OR 'muscle training':ti,ab OR 'functional training':ti,ab OR 'resistance training':ti,ab OR 'endurance training':ti,ab OR 'physical activity':ti,ab OR 'rehabilitation':ti,ab OR 'physical training':ti,ab)<br>#3: ('attitude'/exp OR 'perception':ti,ab OR 'opinion*':ti,ab OR 'perspective':ti,ab OR 'view*':ti,ab OR 'belief*':ti,ab OR 'experience*':ti,ab OR 'needs':ti,ab)<br>#4: ('interview'/exp OR 'focus group':ti,ab OR 'grounded theory':ti,ab OR 'qualitative research':ti,ab OR 'phenomenolog*':ti,ab OR 'narrat*':ti,ab OR 'thematic*':ti,ab OR 'ethnograph*':ti,ab OR 'ethnolog*':ti,ab OR 'qualitative stud*':ti,ab)<br>#1 AND #2 AND #3 AND #4	1456
Date of Search	*1974 to October 19, 2025*	
Cochrane	#1: (stroke OR "post-stroke" OR apoplexy* OR "cerebrovascular accident" OR CVA OR "brain vascular	

Database	Indexed and keyword terms	N
library	accident" OR "cerebral infarction" OR "cerebral ischemia" OR "cerebral hemorrhage" OR "cerebrovascular disease" OR "cerebral embolism" OR "cerebral thrombosis" in Title Abstract Keyword)<br>#2: (exercise* OR "physical activity" OR "physical fitness" OR intervention* OR rehabilit* OR "endurance training" OR "resistance training" OR "functional training" OR "muscle training" OR "aerobic training" OR "gait training" OR "fitness training" OR "conditioning training" OR "core training" in Title Abstract Keyword)<br>#3: (attitude* OR opinion* OR belief* OR need* OR feeling* OR experience* OR perspective* OR view* in Title Abstract Keyword)<br>#4: ("qualitative research" OR interview* OR "focus group" OR "grounded theory" OR phenomenolog* OR narrativ* OR thematic* OR ethnograph* OR ethnolog* in Title Abstract Keyword)<br>#1 AND #2 AND #3 AND #4	894
Date of Search	*1996 to October 19, 2025*	
CINAHL(from EBSCO)	#1: (MH "Stroke" OR stroke* OR post-stroke* OR apoplexy* OR "cerebrovascular accident" OR CVA OR "brain vascular accident" OR "cerebral infarction" OR "cerebral ischemia" OR "cerebral hemorrhage" OR "cerebrovascular disease" OR "cerebral embolism" OR "cerebral thrombosis")<br>#2: (MH "Exercise Therapy" OR MH "Rehabilitation" OR exercis* OR "physical activity" OR "physical fitness" OR intervention* OR rehabilit* OR "endurance training" OR "resistance training" OR "functional training" OR "muscle training" OR "aerobic training" OR "gait training" OR "fitness training" OR "conditioning training" OR "core training")<br>#3: (MH "Attitude" OR MH "Perception" OR attitude* OR opinion* OR belief* OR need* OR feeling* OR experience* OR perspective* OR view*)<br>#4: (MH "Qualitative Research" OR MH "Interviews" OR MH "Focus Groups" OR MH "Grounded Theory" OR interview* OR "focus group*" OR "grounded theory" OR "qualitative research" OR "qualitative study" OR phenomenolog* OR narrativ* OR thematic* OR ethnograph* OR ethnolog*)<br>#1 AND #2 AND #3 AND #4	170
Date of Search	*1988 to October 19, 2025*	
Web of Science	#1: stroke (Topic) or post-stroke* (Topic) or apoplexy* (Topic) or cebrovascular accident (Topic) or brain vascular accident (Topic) or cerebral infarction (Topic) or cerebral ischemia (Topic) or cerebral hemorrhage (Topic) or cerebrovascular disease (Topic) or cerebral embolism (Topic) or cerebral thrombosis (Topic)<br>#2: exercise* (Topic) or fitness training (Topic) or physical activity (Topic) or physical fitness (Topic) or intervention* (Topic) or rehabilit* (Topic) or endurance training (Topic) or resistance training (Topic) or functional training (Topic) or muscle training (Topic) or aerobic training (Topic) or gait training (Topic) or conditioning training (Topic) or core training (Topic)<br>#3:attitude* (Topic) or belief* (Topic) or opinion* (Topic) or need* (Topic) or feeling* (Topic) or experience * (Topic) or perspective* (Topic) or view* (Topic)<br>#4: qualitative research (Topic) or interview* (Topic) or focus group* (Topic) or grounded theory (Topic) or phenomenolog* (Topic) or narrativ* (Topic) or thematic* (Topic) or ethnograph* (Topic) or ethnolog* (Topic)<br>#1 AND #2 AND #3 AND #4	193
Date of Search	*1980 to October 19, 2025*	

Database	Indexed and keyword terms	N
PsycINFO (from EBSCO)	#1: (MH "Stroke" OR stroke* OR post-stroke* OR apoplexy* OR "cerebrovascular accident" OR CVA OR "brain vascular accident" OR "cerebral infarction" OR "cerebral ischemia" OR "cerebral hemorrhage" OR "cerebrovascular disease" OR "cerebral embolism" OR "cerebral thrombosis")	
	#2: (MH "Exercise" OR MH "Rehabilitation" OR exercis* OR "physical activity" OR "physical fitness" OR intervention* OR rehabilit* OR "endurance training" OR "resistance training" OR "functional training" OR "muscle training" OR "aerobic training" OR "gait training" OR "fitness training" OR "conditioning training" OR "core training")	
	#3: (MH "Attitude" OR MH "Perception" OR attitude* OR opinion* OR belief* OR need* OR feeling* OR experience* OR perspective* OR view*)	
	#4: (MH "Interviews" OR MH "Focus Groups" OR MH "Grounded Theory" OR MH "Qualitative Research" OR interview* OR "focus group*" OR "grounded theory" OR "qualitative research" OR "qualitative study" OR phenomenolog* OR narrativ* OR thematic* OR ethnograph* OR ethnolog*)	
	#1 AND #2 AND #3 AND #4	1605
Date of Search	*1982 to October 19, 2025*	
