# Supplementary material for: Meta-synthesis of qualitative studies on home-based exercise rehabilitation experiences among stroke patients: a continuity of care perspective
Source: Front Rehabil Sci. 2026 Mar 4;7:1742902. doi: 10.3389/fresc.2026.1742902 (PMC12995860; doi:10.3389/fresc.2026.1742902)
Supplement: Supplementary file 3 [file Table2.docx]

**Supplementary file 2**

**Table S2 ConQual Grading Details of Included Studies**

| **Study Identification** | **JBI Quality Appraisal (Key Items: 2,3,4,6,7)** | **Dependability Assessment** | **Credibility Assessment** | **Contribution to Synthesized Themes** | **Notes** |
| --- | --- | --- | --- | --- | --- |
| Khoshbakht Pishkhani et al. (2019) | Items 2:Y, 3:Y, 4:Y, 6:U, 7:U → Total Y=3 | 2–3 "Yes" → Downgraded by 1 level (from High to Moderate) | Original findings: 3 clear, 2 plausible → Mixed (clear + plausible) → Downgraded by 1 level | Supports "Gaps in Nursing Guidance" and "Family Support" themes | No researcher bias reported (Item 6=U) |
| Pereira et al. (2021) | Items 2:Y, 3:Y, 4:Y, 6:Y, 7:Y → Total Y=5 | 4–5 "Yes" → No downgrade (remains High) | Original findings: 5 clear → No downgrade (remains High) | Core contributor to "Continuity of Care and Family Support" theme | Full researcher reflection (Items 6=Y, 7=Y) |
| Van Dongen et al. (2021) | Items 2:Y, 3:Y, 4:Y, 6:U, 7:U → Total Y=3 | 2–3 "Yes" → Downgraded by 1 level (from High to Moderate) | Original findings: 4 clear, 1 plausible → Mixed → Downgraded by 1 level | Supports "Self-Regulation Mechanisms" and "Community Support" themes | No researcher-participant relationship described (Item 7=U) |
| Yoshida et al. (2021) | Items 2:Y, 3:Y, 4:Y, 6:U, 7:Y → Total Y=4 | 4–5 "Yes" → No downgrade (remains High) | Original findings: 3 clear, 2 plausible → Mixed → Downgraded by 1 level | Key for "Motivational Fluctuations" in "Self-Regulation" theme | Partial researcher reflection (Item 7=Y, Item 6=U) |
| Chau et al. (2022) | Items 2:Y, 3:Y, 4:Y, 6:U, 7:U → Total Y=3 | 2–3 "Yes" → Downgraded by 1 level (from High to Moderate) | Original findings: 2 clear, 3 plausible → Mixed → Downgraded by 1 level | Contributes to "Discharge Transition Gaps" and "Community Support" themes | No researcher bias disclosure (Item 6=U) |
| Kelly et al. (2022) | Items 2:Y, 3:Y, 4:Y, 6:Y, 7:U → Total Y=4 | 4–5 "Yes" → No downgrade (remains High) | Original findings: 4 clear, 1 plausible → Mixed → Downgraded by 1 level | Supports "Indigenous Patient Transition" in "Nursing Guidance Gaps" theme | Partial researcher reflection (Item 6=Y, Item 7=U) |
| Levy et al. (2022) | Items 2:Y, 3:Y, 4:Y, 6:Y, 7:Y → Total Y=5 | 4–5 "Yes" → No downgrade (remains High) | Original findings: 5 clear → No downgrade (remains High) | Core for "Upper Limb Compliance" in "Self-Regulation" theme | Full researcher bias disclosure (Items 6=Y, 7=Y) |
| Krawczyk et al. (2023) | Items 2:Y, 3:Y, 4:Y, 6:Y, 7:Y → Total Y=5 | 4–5 "Yes" → No downgrade (remains High) | Original findings: 4 clear, 1 plausible → Mixed → Downgraded by 1 level | Contributes to "Group Exercise Preference" in "Community Support" theme | Full methodological transparency (Items 6=Y, 7=Y) |
| Smith et al. (2023) | Items 2:Y, 3:Y, 4:Y, 6:Y, 7:U → Total Y=4 | 4–5 "Yes" → No downgrade (remains High) | Original findings: 3 clear, 2 plausible → Mixed → Downgraded by 1 level | Supports "Peer Accountability" in "Community Support" theme | No researcher-participant impact described (Item 7=U) |
| Zhang et al. (2023) | Items 2:Y, 3:Y, 4:Y, 6:U, 7:U → Total Y=3 | 2–3 "Yes" → Downgraded by 1 level (from High to Moderate) | Original findings: 2 clear, 3 plausible → Mixed → Downgraded by 1 level | Key for "Family Reminders" in "Family Support" theme | No researcher positionality reported (Item 6=U) |

**Notes on ConQual Grading Logic**

**Dpendability (Methodological Rigor):**

Assessed using Items 2, 3, 4, 6, 7 of the JBI Qualitative Research Appraisal Checklist (see 2.8.1).

6/10 studies had 2–3 "Yes" ratings (downgraded by 1 level); 4/10 studies had 4–5 "Yes" ratings (no downgrade).

**Credibility (Findings Validity):**

Based on whether original study findings were "clear" (directly supported by data) or "plausible" (logically inferred but not explicitly stated; see 2.8.2).

All 10 studies included a mix of "clear" and "plausible" findings (36 clear, 12 plausible total; see 3.4), leading to a 1-level downgrade for credibility.

**Final ConQual Score for Synthesized Themes:**

All 4 synthesized themes (Table 4) inherited the "Moderate" score because:

Dependability was downgraded in 6/10 studies;

Credibility was downgraded in all 10 studies (mixed findings);

No theme received "Low" scores (all findings had logical consistency and cross-study support).
